# Villi Inspired Mechanical Interlocking for Intestinal Retentive Devices

**DOI:** 10.1002/advs.202301084

**Published:** 2023-07-14

**Authors:** Durva Naik, Gaurav Balakrishnan, Mahathy Rajagopalan, Xiaozili Huang, Nihar Trivedi, Arnav Bhat, Christopher J. Bettinger

**Affiliations:** ^1^ Materials Science and Engineering Department Carnegie Mellon University 5000 Forbes Avenue, Wean Hall, 3325 Pittsburgh PA 15213 USA; ^2^ Biomedical Engineering Department Carnegie Mellon University 5000 Forbes Avenue, Scott Hall, 4N201 Pittsburgh PA 15213 USA

**Keywords:** digital light processing (DLP) 3D printing, intestinal retention, mechanical interlocking, microstructures, replica molding, villi

## Abstract

Intestinal retentive devices have applications ranging from sustained oral drug delivery systems to indwelling ingestible medical devices. Current strategies to retain devices in the small intestine primarily focus on chemical anchoring using mucoadhesives or mechanical coupling using expandable devices or structures that pierce the intestinal epithelium. Here, the feasibility of intestinal retention using devices containing villi‐inspired structures that mechanically interlock with natural villi of the small intestine is evaluated. First the viability of mechanical interlocking as an intestinal retention strategy is estimated by estimating the resistance to peristaltic shear between simulated natural villi and devices with various micropost geometries and parameters. Simulations are validated in vitro by fabricating micropost array patches via multistep replica molding and performing lap‐shear tests to evaluate the interlocking performance of the fabricated microposts with artificial villi. Finally, the optimal material and design parameters of the patches that can successfully achieve retention in vivo are predicted. This study represents a proof‐of‐concept for the viability of micropost‐villi mechanical interlocking strategy to develop nonpenetrative multifunctional intestinal retentive devices for the future.

## Graphical abstract

Mechanical interlocking of synthetic elastomeric microposts and intestinal villi is studied to develop intestinal retentive devices. Proof‐of‐concept is established through simulations and validated by in‐vitro lap‐shear tests using microfabricated micropost array patches. Computational simulations are utilized to optimize interlocking via design and material parameters, while high‐aspect‐ratio microposts are produced using multistep replica molding with digital light processing 3D printed masters.

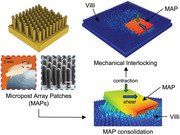

## 1 Introduction

Intestinal retentive devices have applications ranging from long‐term oral drug delivery to indwelling medical devices for monitoring in vivo gastric function and diagnosing gastrointestinal disorders like irritable bowel syndrome , Crohn's disease, intestinal bleeding, and ulcers.^[^ ^1^, ^2^, ^3^, ^4^, ^5^, ^6^, ^7^, ^8^, ^9^, ^10^, ^11^ ^]^ However, there are several challenges to the design and deployment of structures within the small intestine. Persistent gastrointestinal motility subject devices to periodic compressive pressures of ≈20 mmHg and shear stresses of ≈1 N cm^−2^ at frequencies of 0.125–0.3 Hz.^[^ ^12^, ^13^, ^14^, ^15^, ^16^, ^17^ ^]^ Other challenges include rapid mucus turnover (every 24–48 h), enzymatic degradation, and extreme pHs.^[^ ^13^, ^18^, ^19^, ^20^ ^]^

Chemo‐ and mechano‐adhesive approaches have been commonly used to overcome the aforementioned unique constraints and inform designs for intestinal retentive devices (Table S1, Supporting Information). Current chemo‐adhesives include mucoadhesives, such as insulin‐ dimethyl palmitoyl ammonio propanesulfonatepatches,^[^ ^21^ ^]^ in situ gelation techniques such as synthetic epithelium lining,^[^ ^22^ ^]^ or nano/microparticle based systems.^[^ ^23^, ^24^, ^25^, ^26^ ^]^ Chemo‐adhesive technologies however are susceptible to fouling overtime and are rapidly eliminated due to mucus overturn. Alternatively, mechano‐adhesives comprise predominantly of bio‐inspired variations of penetrating microneedles such as hookworm inspired tissue attachment mechanisms,^[^ ^27^ ^]^ Theragrippers,^[^ ^28^ ^]^ barbed microneedles which are inspired by proboscis of spiny‐head worms^[^ ^29^ ^]^ and biphasic swellable microneedles inspired by proboscis of endoparasite Pomphorhynchus laevis.^[^ ^30^ ^]^ While these techniques show some promise in achieving retention in small intestine, deployment of such tissue piercing structures for prolonged periods may lead to bacterial infection.^[^ ^31^ ^]^

**Figure 1:**
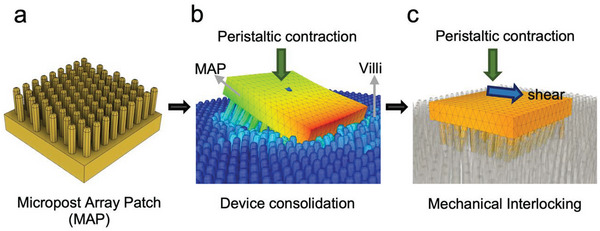
Concept schematic—Mechanical interlocking of intestinal villi and villi‐mimicking synthetic elastomeric microposts. a) Macroscopic device with elastomeric micropost arrays (MAP). b) Villi‐mimicking microposts consolidate within the villi network due to peristaltic contractile pressure. c) Mechanical interlocking of villi and microposts counteracts peristaltic shear and anchors MAP in the small intestine.

Another mechano‐adhesive approach includes friction enhancement between device substrates and intestinal tissue using elastomeric microstructures of aspect ratio ≈1:1. The microstructures were assessed for their ability to clamp endoscopic capsules in the small intestine by Kwon et al.^[^ ^32^, ^33^, ^34^ ^]^ In this study, we evaluate the potential of mechanical interlocking between of high‐aspect‐ratio (≈5:1) elastomeric microposts and the intestinal villi to resist peristaltic shear (**Figure** **1**). Compared to low aspect ratio microstructured devices, we expect high‐aspect‐ratio microposts, with comparable geometry to that of the villi, to induce interlocking with the villi and resist peristaltic shear in the small intestine thereby enhancing its retention time (Figure S1 and Movie S1, Supporting Information). Shear interlocking, a bio‐inspired technique observed in cuticular hairs of beetle wings, has inspired devices capable of reversible detachment.^[^ ^35^ ^]^ While in beetle wings Van der Waals interactions lead to adhesion, in our study we evaluate the ability of collisions to retard movement of microposts and further mechanically interlock within the intestinal villi.

## 2 Results and Discussion

### 2.1 Modeling and Mechanical Simulations of Micropost‐Villi Interlocking

First, we demonstrate the feasibility of mechanical interlocking strategy to prepare intestinal retentive devices through finite element analysis . Porcine intestinal tissue explants rapidly lose mechanical integrity due to lack of blood pressure in the capillaries of the villi (Figure S2, Supporting Information). Hence, as a substitute to ex vivo lap‐shear tests with bio‐mimetic microposts, mechanical simulations were used to predict the behavior of mechanically interlocked devices. Moreover, the use of computational models allowed us to assess the interlocking phenomenon under simulated peristalsis and scan the effects of multiple micropost array patch (MAP) design parameters on interlocking in an economic and timely manner.

**Figure 2:**
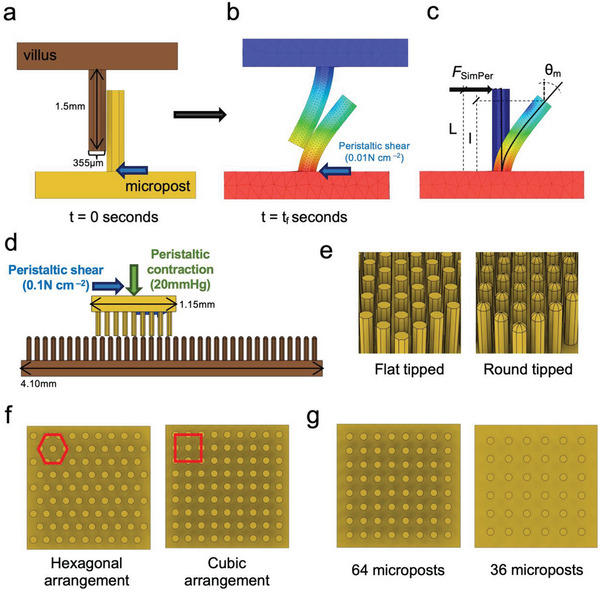
Simulation models—a) MP‐V model. Initial positions of villus and micropost. b) Head‐on micropost‐villus collision under peristaltic shear of 0.01 N cm^−2^. c) Euler–Bernoulli beam theory for large deflection of cantilever beams was used to determine the maximum resistive force (*F*_SimPer_) applied by villus onto the mobile micropost. d) MAP‐VP model. Micropost array patch interlocking with villi under peristaltic contraction (20 mmHg) and shear (0.1 N cm^−2^) conditions. Here, to optimize the computation time, MAP, and villi models are 0.2x the size of MP‐V model. Villi patch is 4x the size of MAPs to mimic the anatomical environment encountered by MAPs in the gut. e) Different tip geometries. f) Arrangement types (*P*_81_). g) Varying pitch of the microposts [edge‐to‐edge spacing varies as 100 µm (*P*_36_), 70 µm (*P*_64_), and 50 µm (*P*_81_)].

Two systems were modeled—a) MP‐V Model: Head‐on collision between a singular micropost and villus under peristaltic shear (**Figure** **2**a,b) and b) MAP‐VP Model: Interaction between MAPs and villi patch (VP) under peristaltic shear and contractile pressure (Figure 2d). Design features of MAPs were varied as followed: flat‐tipped microposts with cubic arrangement (Flat‐Cub), flat‐tipped microposts with hexagonal arrangement (Flat–Hex), round‐tipped microposts with cubic arrangement (Roun–Cub), and round‐tipped microposts with hexagonal arrangement (Roun–Hex) (Figure 2e,g,f). Intestinal peristaltic shears range from 2×10^−8^ to 3.5×10^−4^ N cm^−2 [^ ^36^, ^37^, ^38^, ^39^ ^]^ while the contact forces varies along the gut due to its variable diameter and is estimated to be between 0.9 and 2.9 N cm^−2^.^[^ ^40^, ^41^ ^]^ In the view of this variability, in our simulations we utilized the knowledge of estimated shear stress experienced by bolus, which is ≈1 N cm^−2 [^ ^17^, ^42^ ^]^ as our reference and subjected MP‐V and MAP‐VP simulation models to a peristaltic shear of 0.01 and 0.1 N cm^−2^, respectively. Reduced shear stresses were applied to the models to ensure stability of the simulation environments, allowing us to gather significant and relevant information through them.

In the MP‐V model, the Euler–Bernoulli beam theory for large deflections (Equation (1)) was used to determine the maximum resistive force (*F*_SimPer_) a villus may exert on the mobile micropost (Figure 2c), whereas in the MAP‐VP model, interlocking efficacy of MAPs was quantified by estimating the maximum displacement of MAPs within the VP in the direction of peristaltic shear post definitive consolidation and under sustained peristaltic conditions (*D*_SimPer_) (see the Experimental Section for details).

#### 2.1.1 Simulation Results

**Figure 3:**
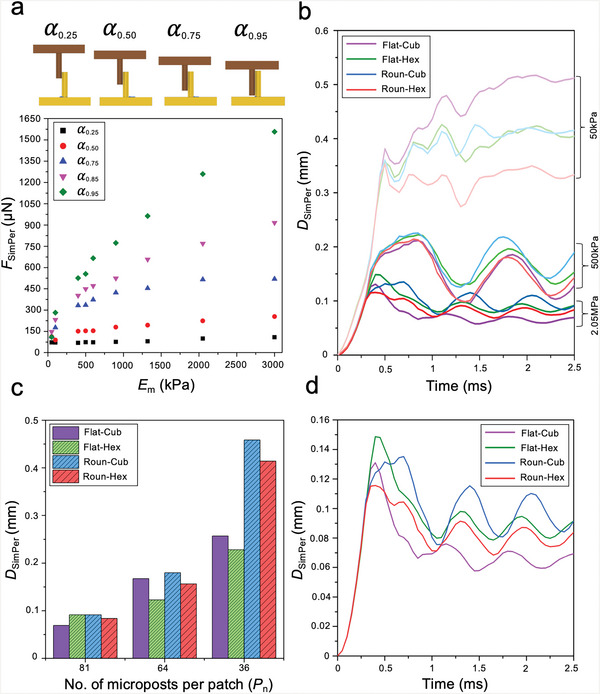
Simulations show that stiffer elastomeric microposts produce greater resistive forces with minimum deformation compared to more compliant elastomers. *F*_SimPer_ increases with the overlap between the micropost and villus. Influence of micropost moduli and array pitch is stronger than the effect of design parameters when microposts dimensions and pitch are comparable to the villi. Such MAPs attain >90% overlap within the villi with jejunal contractive pressure eliminating the need for external insertion mechanisms. a) MP‐V model—Influence of overlapping extent (𝛼) and Young's moduli of microposts (*E*_m_) on maximum resistive force applied by the villus onto the micropost (*F*_SimPer_). b) MAP‐VP—Influence of MAP design and material parameters (*P*_81_). c) Influence of varying pitch on *D*_SimPer_ (*E*_m2.05 MPa_). d) Influence of micropost arrangement and tip‐geometry on maximum displacement in the direction of applied shear (*E*_m2.05 MPa_, *P*_81_), Roun–Hex and Flat‐Cub travel the least within the villi.

The effect of microposts’ Young's moduli (*E*_m_) on maximum resistive force at varying overlapping extents (𝛼) is summarized in **Figure** **3**a. With an increase in *E*_m_ and 𝛼, *F*_SimPer_ ranged from 80 µN (𝛼_0.25_, *E*_m50kPa_)—1600 µN (𝛼_0.95_, *E*_m3MPa_). At 𝛼_0.25_, microposts with *E*_m_ ≥ 900 kPa bent negligibly (5–0.4 µm) while achieving an *F*_SimPer_ comparable to microposts with lower stiffnesses that significantly deformed (500–10 µm). These observations suggest that stiffer microposts produce higher resistive forces through micropost‐villus collision with minimum deformation compared to compliant microposts with lower stiffnesses. Further, as anticipated, it was observed that 𝛼 and *F*_SimPer_ are proportional since with 𝛼 the distance to the contact point decreases and bending angle of the micropost increases.

*D*_SimPer_ of *E*_m50kPa_ MAPs containing 81 microposts per patch (*P*_81_) and with varying design features decreased by ≈62% and ≈79% on an average when MAP stiffness increased by 10x and 40x, respectively. Increasing MAP stiffness also induced an underdamped harmonic oscillatory behavior in the MAP‐VP interlocking (Figure 3b). Upon increasing the areal density of the MAP, *D*_SimPer_ (*E*_m2.05 MPa_) decreased—an anticipated result based on the increased micropost‐villi collisions (Figure 3c; and Figure S3 and Movie S2, Supporting Information).

Further, MAPs with flat‐tipped microposts in *P*_64_ and *P*_36_ designs consistently displayed lower *D*_SimPer_ compared to their round‐tipped counterparts (Movies S2 and S3, Supporting Information), i.e., *D*_SimPer_ Flat‐cub < Roun‐cub and *D*_SimPer_ Flat‐Hex < Roun–Hex. This behavior was attributed to the additional surface area provided by the flat‐tips which increases the frictional resistance and bolsters the footing of the microposts onto the substrate of the villi patch. The impact of this design difference on MAP‐VP interlocking is patent in *P*_36_ interlocking systems (Figure S3, Supporting Information) as round‐tipped MAPs (*E*_m2.05 MPa_) compared to their flat counterparts cease to portray the underdamped oscillatory behavior and flat‐tipped systems display a decrease of *D*_SimPer_ of 78% and 81% in the cubic and hexagonal arrangements, respectively. It was also observed that hexagonal arrangement of microposts in *P*_64_ and *P*_36_ designs compared to cubic consistently delivered reduced *D*_SimPer_. This observation was associated with the increased intra‐micropost interactions that may be induced by the hexagonal arrangement of microposts and cubic arrangement of villi.

In case of *P*_81_ MAP designs, the spacing between the microposts (50 µm) is comparable to pitch of villi patch (40 µm). Hence factors like ease of penetration into the villi patch and overlapping extent may also influence interlocking efficacy of the MAPs. Overall, here the difference within the *D*_SimPer_ values for various design parameters lied within ±0.01 mm and, Flat‐Cub and Roun–Hex MAPs (*E*_m_2.05 MPa) were least displaced under the applied shear (70 and 80 µm, respectively) (Figure 3d). The ratio of damping constants of Roun–Hex: Flat‐Cub was estimated to be 1.03 signifying that Roun–Hex MAPs achieve their equilibrium position faster than Flat‐Cub MAPs and can thereby consolidate more quickly within the villi under constant peristaltic shear.

Overall, these observations indicate that MAP design features may influence mechanical interlocking of MAPs within the villi. Additionally, all MAPs achieved an overlapping extent of >90% within the villi solely due to the simulated jejunal contractile pressure. These results suggest that compliant micropost arrays with a size and spacing comparable to natural villi can fully integrate within villi thereby obviating the need for supplementary insertion mechanisms.^[^ ^34^, ^43^ ^]^

### 2.2 In Vitro Analysis of Mechanical Interlocking between MAPs and Artificial Villi

We experimentally validate the in silico models by microfabricating micropost array patches via a robust, cost‐effective, and adaptable multistep replica molding technique which employs digital light processing (DLP) 3D printed molds and further conducting in vitro lap‐shear experiments between artificial villi and MAPs on customized equipment. Through these studies, we gain insight into the influence of material and design parameters of MAPs on their efficiency to interlock within the villi under shear.

**Figure 4:**
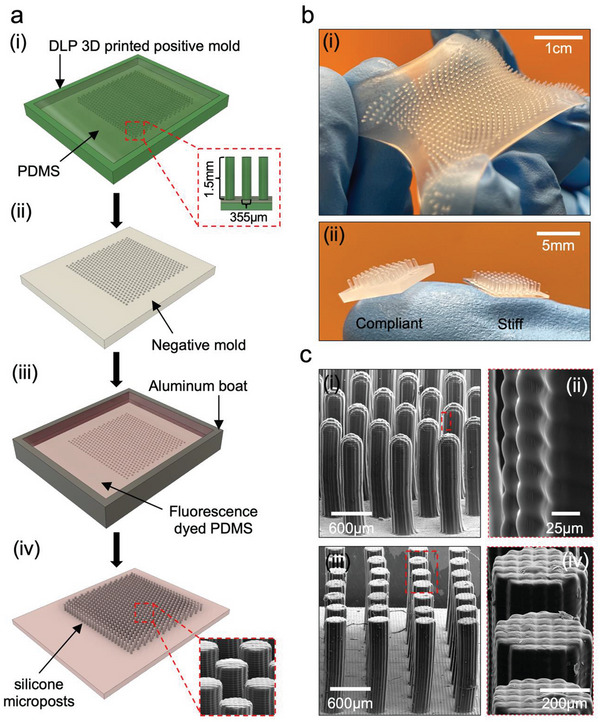
Artificial villi and MAPs fabricated by replica molding from masters fabricated via digital light processing (DLP) 3D printing a) Microfabrication steps: (i) DLP 3D printed positive molds. (ii) Negative mold replica molded using Sylgard‐184, followed by oxygen plasma cleaning and O/N silanization treatment. (iii) Replica molding elastomeric microposts using mixtures of Ecoflex‐0010 and Sylgard‐184 for desired MAP/villi patch material properties. (iv) Demolding silicone‐based microposts. b) (i) Artificial villi patch (edge‐to‐edge spacing = 200 µm, 4 cm^2^, *E*_v50kPa_) (ii) Micropost Array Patches (25 mm^2^); Compliant Ecoflex‐0010 based microposts (*E*_m50kPa_) (left), Stiff Sylgard‐184 based microposts (*E*_m2.05 MPa_) (right). c) SEM imaging of fabricated microposts (i) MAPs with round‐tipped microposts arranged hexagonally (Roun–Hex) (ii) MAPs with flat‐tipped microposts arranged cubically MAPs (Flat‐Cub) (edge‐to‐edge spacing = 350 µm, aspect ratio 4.2: 1, L = 1.5 mm). (iii, iv) Surface detail of microposts.

MAPs (5×5 mm^2^) and artificial villi (2×2 cm^2^) were fabricated by a multistep replica molding technique using DLP 3D‐printed molds (**Figure** **4**a).^[^ ^44^, ^45^ ^]^ Compared to other microfabrication methods, such as soft‐lithography,^[^ ^46^, ^47^, ^48^ ^]^ laser cutting,^[^ ^49^ ^]^ and electrical discharge machining,^[^ ^50^, ^51^ ^]^ replica molding using DLP 3D‐printed molds is a cost‐effective strategy to fabricate high‐aspect‐ratio soft microposts of arbitrary dimensions and with high fidelity (Figure 4c).

**Figure 5:**
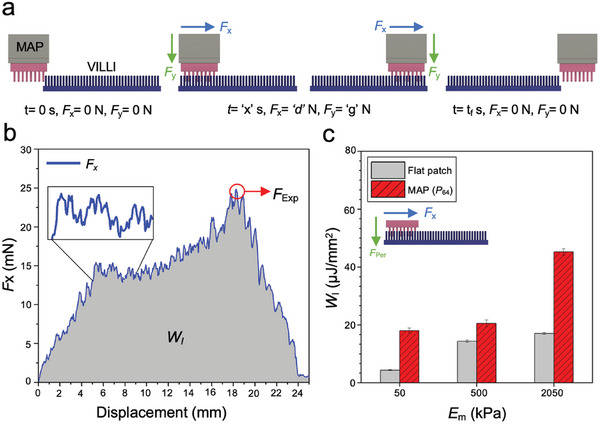
In vitro lap‐shear tests a) schematic of lap‐shear testing procedure. b) Force‐displacement curve of MAP with *E*_m2.05 MPa_ interlocking with artificial villi at overlap of 𝛼_0.5_. Interlocking was characterized by measuring work of interlocking [*W*_I_ (shaded area)] and maximum interlocking force (*F*_Exp_). Slip‐stick phenomenon confirmed the occurrence of mechanical interlocking between the artificial villi and microposts. c) Experimental work of adhesion (*W*_A_) of flat patches increases due to mechanical interlocking of microposts with the villi (*n* = 3).

Using fabricated MAPs/villi patches, work of interlocking (*W*_I_) and maximum interlocking force of MAPs‐Villi interlocking (*F*_Exp_) were measured using a customized lap‐shear test bed (**Figure** **5**a; and Figure S4, Supporting Information). Slip‐stick events were observed from force‐distance measurements as MAP microposts moved through the villi thus supporting the anticipated mechanical interlocking mechanism (Figure 5b).^[^ ^52^, ^53^ ^]^ Shear adhesion of flat patches on villi amplified upon the introduction of microposts due to induced interlocking (Roun–Hex, *P*_64_); depending on the moduli of the patch, increase in adhesion work (*W*_A_) due to mechanical interlocking ranged from 40% to 300% under a constant preload of 2.4 mN mm^−2^ (Figure 5c). This preload value of 2.4 mN mm^−2^ (*F*_Per_) was selected as it corresponds to the in vivo intestinal contractile pressure of 20 mmHg.^[^ ^54^ ^]^

**Figure 6:**
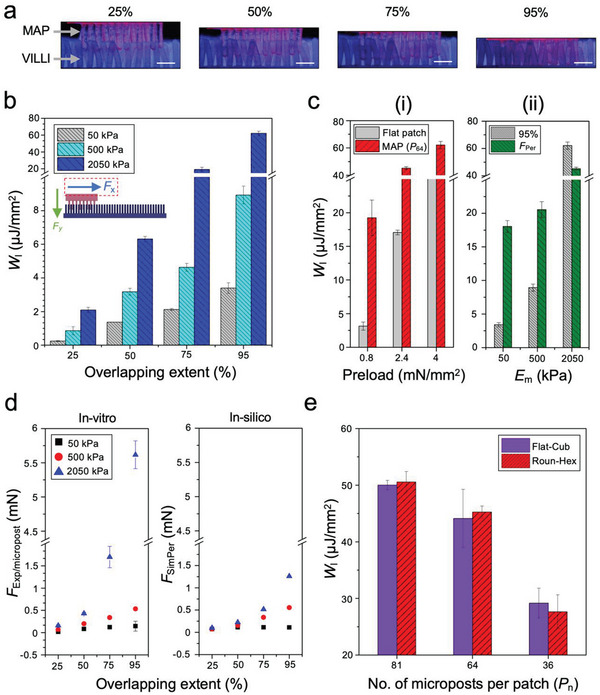
In vitro analyses were consistent with simulation outcomes thereby substantiating computational simulations as a tool for parametric studies to predict the in vivo mechanical interlocking of MAP‐based devices. a) Micrographs of the experimental test‐bed indicating varying overlaps between microposts and artificial villi (scale bar = 1 cm). b) Increased work of interlocking was observed with increased MAP stiffness and overlapping extent. c) Linear relationship between preload and work of interlocking is observed. d) Maximum interlocking force per micropost from experiment and simulation showed similar trends. e) Increasing MAP pitch increased interlocking work, as expected and observed in simulations (*n* = 3).

*W*_I_ of MAPs (Roun‐Hex, *P*_64_) were measured as a function of moduli and micropost‐villi overlap (**Figure** **6**a). Experimental trends in *E*_m_ and 𝛼 were consistent with mechanical simulations (Figure 6b). Further, the extent of micropost‐villi overlap scaled with preload (Figure S5, Supporting Information) and tests showed that larger preload increases *W*_I_ (Figure 6ci). Preload‐dependency on interlocking has been reported in literature for bio‐inspired systems that rely on Van der Waals forces (beetle‐wings)^[^ ^35^ ^]^ and in microhooks (climbing plants).^[^ ^55^ ^]^ It was also noted that stiffer MAPs (*E*_m2.05 MPa_) require preload >*F*_Per_ to achieve 𝛼>95%, while more complaint MAPs (*E*_m50/500 kPa_) obtain 𝛼>100% under *F*_Per._ Under *F*_Per,_ compliant MAPs *E*_m50kPa_ and *E*_m500kPa_ display ≈400% and ≈100% increase in *W*_I_, respectively, compared to 𝛼_0.95_ (Figure 6cii). This indicates that stiffness of microposts affects not only the resistive force from micropost‐villus collisions but also the ability for MAPs to penetrate with villi under a given preload.

We compared the max resistive forces per micropost obtained from in silico and in vitro analyses (e.g., *F*_SimPer_ and *F*_Exp/micropost_). *F*_Exp/micropost_: *F*_SimPer_ varied from 0.85 ± 0.467 (*E*_m50kPa_) to 2.82 ± 1.32 (*E*_m2.05 Mpa_) with a general trend of increased deviation with greater *E*_m_ and 𝛼 (Figure 6d). This difference can be attributed to the contribution of frictional resistance during in vitro analyses which cannot be eliminated due to the microtextured surface of the microposts (*R*_a_ = 25µm) (Figure 4c). Friction‐enhancement with micropatterned structures for intestinal adhesion has proved beneficial.^[^ ^33^ ^]^ We hypothesize that texture induced friction will only enhance mechanical interlocking and improve resistance to movement due to peristalsis in vivo. Other factors that contribute to this nonlinear interlocking‐assisted adhesion by MAPs, which are unaccounted in MP‐V models, include intra‐micropost collisions and the effect of multiple simultaneous micropost‐villi collisions. The synergistic influence of these factors is also noted in MAP‐VP simulation models where MAPs, irrespective of the moduli and design, can counteract 10 times the shear applied to the MP‐V models (0.01 N cm^−2^) via interlocking and immobilize within the villi (Movie S4, Supporting Information), whereas a single micropost‐villus collision cannot resist the peristaltic shear (Movie S5, Supporting Information). We also observed an increase in the interlocking work with greater MAP pitch, an anticipated result which was also learned via simulations (Figure 6e). Overall, results from in silico modeling of mechanical interlocking were consistent with the in vitro experimental results. Accurate computational simulations substantiate this tool to design and optimize the device for in vivo retention of MAP‐based devices under peristaltic conditions.

### 2.3 MAP Design and Material Analysis to Optimize In Vivo Retention

We use in silico modeling to predict the optimal MAP parameters for in vivo device retention. Through in silico analysis of mechanical interlocking, we learned that MAP stiffness and design parameters such as tip‐geometry, pitch, micropost layout, etc., affect MAP's resistance to movement when mechanically interlocked with the villi. In this substudy, we aimed to maximize *F*_SimPer_ by virtue of these parameters and thereby optimize in vivo residence time of MAP‐based devices.

**Figure 7:**
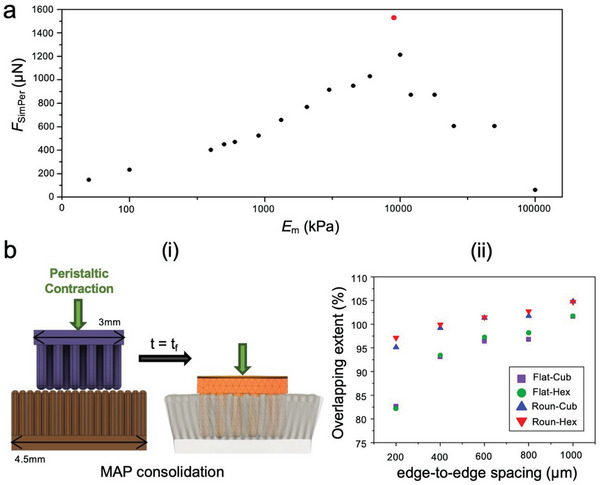
MAP material and design parameters to optimize in vivo residence time of fabricated devices. a) Maximum resistive force peaks when *E*_m_ = 9 MPa (𝛼_0.85_). b) (i) Schematic of MAP (3×3 mm^2^) consolidating within villi under constant peristaltic contraction. (ii) Increasing spacing between the microposts increases the extent of overlap. MAPs with round‐tipped microposts arranged in hexagonal pattern with edge‐to‐edge spacing of 400 µm maximize the number of microposts while attaining >100% thereby optimizing the cumulative resistive force.

Using the MP‐V model, we observed that *F*_SimPer_ peaks when *E*_m_ is 9 MPa (𝛼_0.85_). Deformation of microposts with *E*_m_ > 9 MPa is negligible (6 µm–6 nm) since their movement is unobstructed by presence of the villus (**Figure** **7**a). As established previously, *F*_SimPer_ increases with overlapping extent between microposts and villi. The villi of our small intestine are densely arranged in a close‐packed geometry with ≥40 villi per mm^2^.^[^ ^56^ ^]^ We anticipate jejunal contractions to insert the micropost arrays into the villi and achieve an overlapping extent of >100%. A wider distribution of microposts on MAP may enhance its penetration but it will result in reduced micropost‐villi collisions thereby decreasing the overall resistance to peristaltic shear via mechanical interlocking. To study the influence of MAP design parameters on the extent of penetration under constant jejunal contractile pressure, we modeled villi patches (asp. ratio ≈6:1) to maximize the pitch of the villi (edge‐to‐edge spacing = 9 µm) (Figure 7bi). We observed that an increase in spacing between the microposts led to greater overlap, and round‐tipped microposts achieved >95% overlap irrespective of the arrangement. MAPs with round‐tipped microposts arranged in hexagonal pattern with edge‐to‐edge spacing of 400 µm maximized the number of microposts and achieved >100% overlap (Figure 7bii).

While this research serves as a proof‐of‐concept for the mechanical interlocking between villi and synthetic microposts to combat constant peristaltic shears, influence of factors such as intestinal fluid volumes and periodic peristaltic contractions have not been explored in this study. Notably, studies performed by Mosgaard et al., where they examined the adhesion of cylindrical microcontainers (with a diameter of 300 µm and aspect ratio ≈1) in ex vivo perfusion models under a perfusion rate of 1.55 mL min^−1^, indicated that microcontainers exhibit good adhesion properties and also reported that taller cylindrical microcontainers travel lesser distance within the intestinal mucosa compared to shorter cylinders.^[^ ^57^ ^]^ This suggests that our devices, given the size of the cylinders is similar to the size of our device microposts, may also be effective in countering intestinal fluid volumes. We further explored the effect of intestinal mucosa on mucoadhesion of our devices. Ex vivo lap‐shear tests conducted between the microposts (*E*_m_ = 2.05 MPa, *P*_81_, and preload of 2.4 mN mm^−2^) and intestinal tissues revealed a frictional force of 1.375±0.07 mN mm^−2^, which is comparable to other intestinal retention technologies (Table S1, Supporting Information). This implies that, following device consolidation, the mucus layer may contribute to an enhanced resistance to shear due to the frictional drag force experienced by the microposts as they traverse the mucus layer.^[^ ^33^ ^]^ Although, the mucus may create slippery contacts between the microposts and villi, our simulations, which take this factor into account, confirm the feasibility of mechanical interlocking even in such conditions.

It should be noted that in these ex vivo tests, the effect of mechanical interlocking between the villi and microposts do not contribute to adhesion with the tissue due to the loss of mechanical integrity of villi ex vivo. Nonetheless, the ex vivo lap‐shear tests suggest the possibility of synergistic retention capabilities in vivo and therefore, to fully explore the potential of interlocking systems, it is crucial to conduct in vivo studies in the future. Overall, our findings highlight the promising nature of mechanical interlocking systems and emphasize the need for further exploration in this field.

## 3 Conclusion

This study confirms the viability of using mechanical interlocking between high‐aspect‐ratio synthetic elastomeric microposts and intestinal villi as a mechanism for constructing intestinal retentive devices. This was demonstrated through a combination of mechanical simulations and in vitro validation via lap‐shear tests using microfabricated biomimetic microposts. The results of these experiments offer valuable insights into the impact of design and material parameters on interlocking and demonstrate the usefulness of computational simulations as a tool for investigating soft interlocking mechanisms. Elastomers with *E* ≈9 MPa provide optimal resistance to peristaltic shear. The implementation of round‐tipped microposts, with an aspect ratio of 4.2: 1 and diameter of 355 µm arranged in a hexagonal pattern with a 400 µm interpost spacing, has the potential to result in over 100% overlapping with natural villi during jejunal contraction, thus minimizing the need for complex active actuating systems for in vivo deployment.

This research provides a crucial step toward development of customized interlocking devices using in silico modeling.^[^ ^58^ ^]^ Further, the elastomeric nature of these microposts can be used to prepare flexible, expandable structures that can adapt to the folds and contours of the small intestine to improve retention.^[^ ^59^, ^60^, ^61^ ^]^ The use of biodegradable and mucoadhesive materials^[^ ^21^, ^62^, ^63^ ^]^ to create these microposts can be explored to regulate the residence time of these devices for use in various potential applications including: oral drug delivery of macromolecules;^[^ ^13^, ^64^, ^65^ ^]^ real‐time gut monitoring; diagnostic devices^[^ ^66^, ^67^, ^68^, ^69^ ^]^; neural modulation devices.^[^ ^70^ ^]^

## 4 Experimental Section

### Design and Modeling of Mechanical Simulations

Dynamic event simulations were performed on Autodesk Fusion 360. Micropost and villus were designed as octagonal prisms with equivalent dimensions and aspect ratio of 4.2:1. For MP‐V model, the micropost (length 1.5 mm) was joined to a device fragment (3×3 mm^2^) and constrained to move in the direction of applied shear of 0.01 N cm^−2^, whereas the villus was completely fixed. Separation contact model was applied and coefficient of friction between points of contact was set to 0.04 to mimic the slippery nature of the gut. Since the modulus of villus is not reported in literature, modulus of the intestine was referenced and set it as 50 kPa .^[^ ^71^ ^]^ Moduli of microposts (*E*_m_) was varied from 50 kPa to 1 GPa depending on the analyses. Poisson's ratio was set to 0.49 since microposts and villi were held as elastomeric entities. 25 552 parabolic mesh elements were used for calculations with a fine mesh size of 0.05 mm where convergence was achieved. Each study was performed for a total event duration (TED) of 0.01 s (Figure S6a, Supporting Information).

For MAP‐VP model, microposts and villi were designed 0.2 times the size of MP‐V model to optimize the simulation computation time. Size of the VP (4×4 mm^2^) was set 4 times that of MAPs (1 mm^2^). Villi were designed with rounded tips, arranged in a cubic fashion with ≈81 villi per mm^2^ [edge‐to‐edge spacing = 40 µm (*S*_40_), *P*_1296_]. MAP designs were permutations of the following features—pitch per patch (*P*_n_), i.e., *P*_81/64/36_ corresponding to *S*_50/70/100_, arrangement of microposts on the patch, i.e., cubic, or hexagonal and, tip‐geometry—round or flat. Material characteristics of the MAPs and Villi were equivalent to the MP‐V model. Here, MAPs were constrained to move in the direction of the applied shear (0.1 N cm^−2^) and contractile pressure (20 mmHg). To optimize computation time, in this model 263 052 linear mesh elements were used with a mesh size of 0.0857 mm and performed simulations for a TED of 0.0025 s. Observed trends remained the same with varying mesh size (Figure S6b, Supporting Information).

Models for in vivo optimization were designed using MAPs (3×3 mm^2^) with micropost features analogous to the MP‐V model and modulus of 500 kPa. Villi patches (4.5×4.5 mm^2^) with round‐tipped villi with aspect ratio of ≈6:1 (length 1.5 mm) were arranged in a close‐packed cubic fashion (*S*_9_, *P*_324_). MAPs with Flat‐Cub, Flat‐Hex, Roun‐Cub, Flat‐Hex features, and *S*_200‐1000_ were modeled. They were allowed to move vertically in the direction of applied contractile pressure (20 mmHg), while the villi patch was fixed. Other properties of this model were the same as the former models. Here, 463 809 linear mesh elements with a mesh size of 0.214 mm were used and simulations were performed for a TED of 0.01s.

### Mathematical Modeling to Determine Maximum Resistive Force Using MP‐V Models

To estimate the maximum resistive force exerted by the villus on the micropost during peristaltic shear (Figure 2b,c), the theory was employed of large deflection of cantilever beams developed by Bisshop and Drucker.^[^ ^72^, ^73^ ^]^

The decision to utilize the theory of large deflections was based on the simulations which demonstrated that the deformation of the micropost (in the direction of the applied force) resulting from the collision was comparable to the length of the micropost.

In order to employ this theory and estimate *F*_SimPer_, the following assumptions were made: 1) The modeled micropost was considered to be a linear‐elastic cylinder, 2) The micropost was assumed to be anchored to a rigid substrate, 3) The maximum resistive force *F*_SimPer_ was estimated at the time step of the collision just before the micropost slid past the villus and lost contact (Movie S5, Supporting Information), 4) *F*_SimPer_ was assumed to be a concentrated force applied at the tip of the free end and perpendicular to the micropost, resulting in a displacement equivalent to the maximum deformation *“l”* of the tip in the direction parallel to the micropost. The value of *“l”* was obtained from the simulation output (Figure 2c). The following relationship was used

(1) $$F_{\mathrm{SimPer}}\approx \frac{2E_{m}Isin\theta_{m}}{l^{2}}$$

Here, “*θ*_m_” represents the maximum angle of rotation of the deflection curve at the free end, obtained from the simulation output. *E*_m_ is the Young's modulus of the micropost, and “ I” is the moment of inertia of the cross‐sectional area of the micropost about the axis of bending. It is important to note that due to the assumption of the micropost being anchored to a rigid substrate, Equation (1) overestimates the value of the resistive force. This solution does not consider the deformation of the elastic substrate to which the elastomeric microposts are attached. Such factors include shear contributions and base tilting contributions.^[^ ^74^ ^]^ Therefore, the calculated *F*_SimPer_ provides an approximate value of the maximum resistive force.

### Design of Fabricated Microposts Array Patches and Artificial Villi

Mucosa of the gut comprises villi and lubricious mucus gel that coats its lumen to enable passage of the bolus. To mimic mechanical integrity of living villi and eliminate unwanted contributions of frictional drag forces due to mucus‐microposts interactions, lap‐shear studies were conducted with artificial villi. Dimensions of human intestinal villus vary dramatically across the gut (200 µm < *L* < 1500 µm and 60 µm < *d* < 500 µm).^[^ ^56^, ^75^ ^]^ In view of this variability, an aspect ratio of 4.2:1 (*L* = 1.5 mm) was set to prepare the microposts of MAPs and artificial villi. ≈3 villi per mm^2^ (round‐tipped, *E*_v50kPa_) were cubically arranged with an edge‐to‐edge spacing of 200 µm and cross‐sectional area of 2×2 cm^2^ (*P*_1296_) to prepare the artificial villi patch (Figure S7a, Supporting Information). MAPs with cross sectional area of 5×5 mm^2^ were fabricated with varying edge‐to‐edge spacing (250, 350, and 500 µm, i.e., *P*_81_, *P*_64_, and *P*_36_), combinations of cubic/hexagonal arrangement and tip geometry (Figure S7a, Supporting Information).

### DLP Facilitated Multistep Replica Molding Process

Positive PDMS based master molds with desired design parameters were 3D printed with Direct Light Processing (DLP) technique with the Microfluidics 3D printer Mii Ultra100 (CADWorks3D) using master mold resin for PDMS (resinworks3D). Autodesk Fusion360 was used to design and prepare the STL files required for 3D printing. Dimensions of the microposts were optimized to address shrinkage post UV curing and achieve desired aspect ratio of ≈4.2:1 (*L* = ≈1.5 mm, *d* = ≈355 µm) (Tables S2 and S3, Supporting Information). STL files were sliced with Utility software (layer thickness = 30 µm, curing time = 3.5 s). Post printing, molds were carefully placed in a crystallizing dish containing isopropyl alcohol (IPA) (Pharmco, Greenfield Global, CT) and triple washed (20 min each) under ultrasonification (Branson 5800 ultrasonic cleaner, 40 kHz) until clear IPA was achieved. Molds were then air‐dried overnight, and further UV cured (Professional Cure Zone, Creative CADWorks Preset C) for a total of 40 min on each side (Figure S7b, Supporting Information).

Soft PDMS based negative molds were then replica molded with the cured 3D printed molds. Standard 10:1 mixture of Sylgard‐184 (Dow Corning, MI) was poured into the positive molds and then degassed in a vacuum desiccator. The mixture was cured at 75 °C for 90 min in an oven (VWR, Symphony vacuum oven) and the negative mold was further demolded. Post demolding, the molds were further cured for 30 min at 100 °C. The negative molds were silanized using the procedure described by Deng et al.^[^ ^76^ ^]^ Briefly, the negative molds were first treated with oxygen plasma (Plasma cleaner, Harrick plasma) for 90 s under 260 mTorr. The treated molds were then promptly placed in a vacuum desiccator and silanized with trichloro (1H,1H,2H,2H‐perfluorooctyl) silane at 60 mTorr for 24 h. Silanized molds were then baked at 100 °C in the oven for 45 min and then stabilized at room temperature for 60 min (Figure S7c, Supporting Information).

MAPs and villi patches were replica molded using the silanized negative molds with preferred PDMS formulations (Table S4, Supporting Information).^[^ ^77^, ^78^, ^79^ ^]^ The mixture was poured into the negative mold which was secured in an aluminum boat and degassed at 60 mTorr in a vacuum oven for 30 min. PDMS was then cured in the oven. Post curing, the assembly was immediately placed in a −20 °C freezer (VWR) for 5 min, and then in a 75 °C oven for 5 min. This cycle was repeated twice before letting the assembly reach room temperature. MAPs (Roun‐Hex/Flat‐Cub) and artificial villi patches were then demolded from negative molds. To prepare the villi, Ecoflex‐0010 (Dow Corning, MI) with a moduli of 50 kPa was used to mimic mechanical properties of the intestinal villi.^[^ ^79^ ^]^

### Treatment of MAPs and Artificial Villi for In Vitro Analysis

Fabricated items were washed in isopropyl alcohol for 5 min under ultrasonication and then air‐dried. Dried patches were secured to a glass slide. Microbumps (*R*_a_ = 25 µm) were observed on the surface of the microposts suggesting that frictional contribution to adhesion cannot be eliminated in vitro (Figure 4c). A light coating of silicone mold release (Smooth‐On mold release spray, PA) was sprayed onto the patches prior to the in vitro tests to minimize the factor of frictional resistance in adhesion and to even out the effects of tackiness on adhesion imparted by soft PDMS materials. The spray was applied as per the instructions from the manufacturers. Briefly, the spray was held 30 cm away from the patches and a single light mist coating was sprayed onto the patches. The patches were then allowed to sit for 10 min to let the spray dry on the surface of the patches.

### SEM Characterization of Microposts

Samples were mounted onto aluminum stubs and coated with an 8 nm layer of platinum using a sputter coater (EMS Q300T D plus). Surface topography images were obtained with Quanta 600 GEF scanning electron microscope at an accelerating voltage of 20 kV.

### Measurement of Interlocking Properties In Vitro

Lap‐shear tests were performed on a custom‐built test bed equipped with a six‐axis force/torque transducer (Nano‐17 Titanium, ATI Industries). Lap‐shear tests with MAPs, instead of singular micropost‐villus systems, were performed due to the limitation of the equipped transducer to accurately measure forces below 1 mN (force resolution of ≈3 mN). The measuring system comprised a DAQ device with multiple BNC inputs (DAQ PS/IFPS box), BNC interface box (9105‐BNC‐2), and terminal block with BNC inputs connected to a computer running the DAQ software (Figure S4, Supporting Information). ATI DAQ automation server was used to set the data acquisition frequency to 10^5^ Hz at an averaging level of 2000 for data collection. Moving platforms comprised of horizontal and vertical motorized stages (MFA‐CC, Newport) coordinated by a motion controller (ESP301, National Instruments). Custom LabVIEW VI was developed to set interaction velocity and to record displacement of MAPs within the villi. Custom made aluminum parts were attached to the motorized stages to fix MAPs (L‐angled attachment on the vertical axis) and fabricated villi (horizontal axis). Platforms were further equipped with ±5° goniometers (M‐GON40‐L Newport) to ensure planarity prior to testing. Ultraviolet lap and Dino‐lite microscope were utilized to assess interlocking in real time and set the overlapping extent prior to testing.

MAPs and artificial villi were secured on their respective platforms with double sided foam tape. MAPs (top‐fixture) were initially lowered to align microposts and villi tips and, the *z*‐axis displacement was set to zero. MAPs were further lowered at a speed of 0.325 mm s^−1^ until desired overlapping extent/preload was achieved. Every test began with MAPs situated outside the villi patch to ensure load biasing removed false forces. Villi (bottom fixture) was then moved at a speed of 0.2 mm s^−1^. Maximum interlocking force (*F*_Exp_) and work of interlocking (*W*_I_) were obtained from force‐displacement curves.

### Statistical Analysis

Simulation results were reported as is and in vitro lap‐shear test results were averaged over 3 cycles and reported as mean ± standard deviation.

## Conflict of Interest

The authors declare no conflict of interest.

## Supporting information

Supporting Information

Supplemental Movie 1

Supplemental Movie 2

Supplemental Movie 3

Supplemental Movie 4

Supplemental Movie 5

## Data Availability

The data that support the findings of this study are available from the corresponding author upon reasonable request.
