# Supplementary material for: Villi Inspired Mechanical Interlocking for Intestinal Retentive Devices
Source: Adv Sci (Weinh). 2023 Jul 14;10(30):2301084. doi: 10.1002/advs.202301084 (PMC10602537; doi:10.1002/advs.202301084)
Supplement: Supplementary file 1 — Supporting Information [file ADVS-10-2301084-s006.pdf]

ADVANCED SCIENCE
Open Access

Supporting Information

for *Adv. Sci.*, DOI 10.1002/advs.202301084

Villi Inspired Mechanical Interlocking for Intestinal Retentive Devices

Durva Naik, Gaurav Balakrishnan, Mahathy Rajagopalan, Xiaozili Huang, Nihar Trivedi, Arnav Bhat and Christopher J. Bettinger**

Copyright WILEY-VCH Verlag GmbH & Co. KGaA, 69469 Weinheim, Germany, 2018.

Supporting Information

Villi Inspired Mechanical Interlocking for Intestinal Retentive Devices

Durva Naik, Gaurav Balakrishnan, Mahathy Rajagopalan, Xiaozili Huang, Nihar Trivedi, Arnav Bhat, Christopher J. Bettinger**

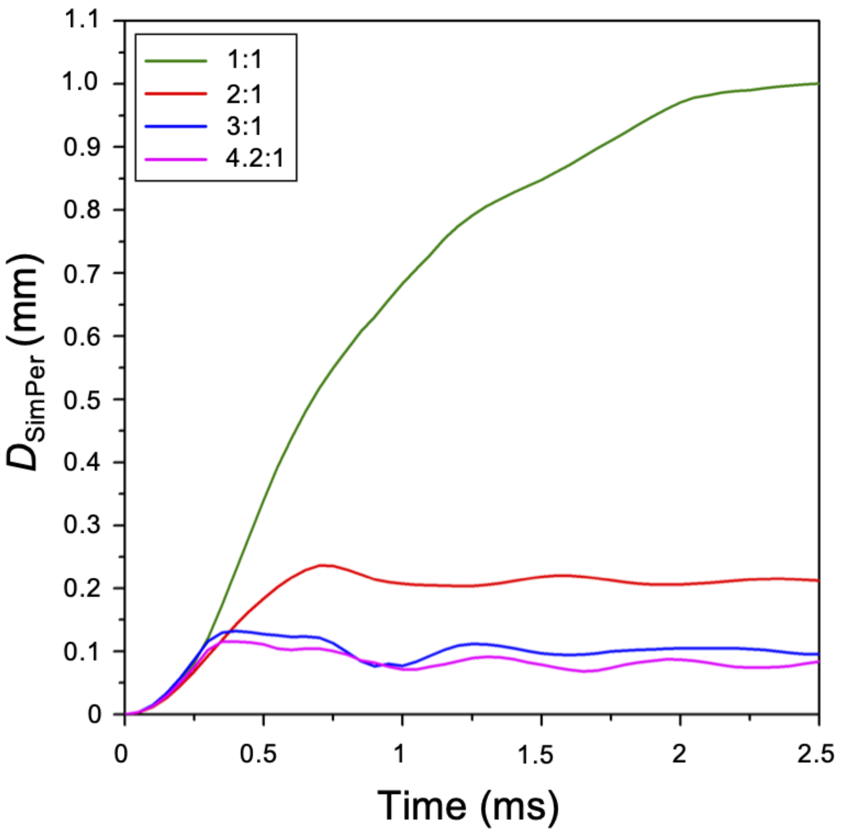

Figure S1: Maximum displacement curves of devices with microposts with different aspect ratios. It is observed that microposts with dimensions comparable to the villi can mechanically interlock with the villi which can enhance retention time of devices in the small intestine. Here, propagation of devices with high-aspect-ratio microposts decreases ~10 times compared to low aspect ratio devices (Movie S1).

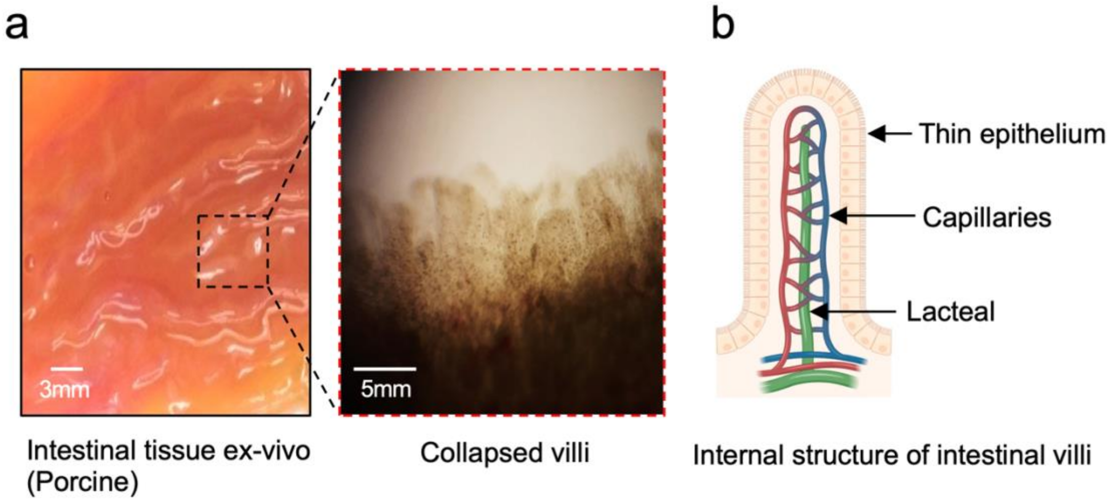

Figure S2: Ex-vivo porcine villi morphology a) Fresh harvested mucosa from porcine small intestine. Intestinal tissue observed under optical microscope reports collapsed villi. b) Structure of villus; intestinal villi collapse *ex-vivo* due to lack of blood pressure in the capillaries thereby loosing mechanical integrity (created with BioRender).

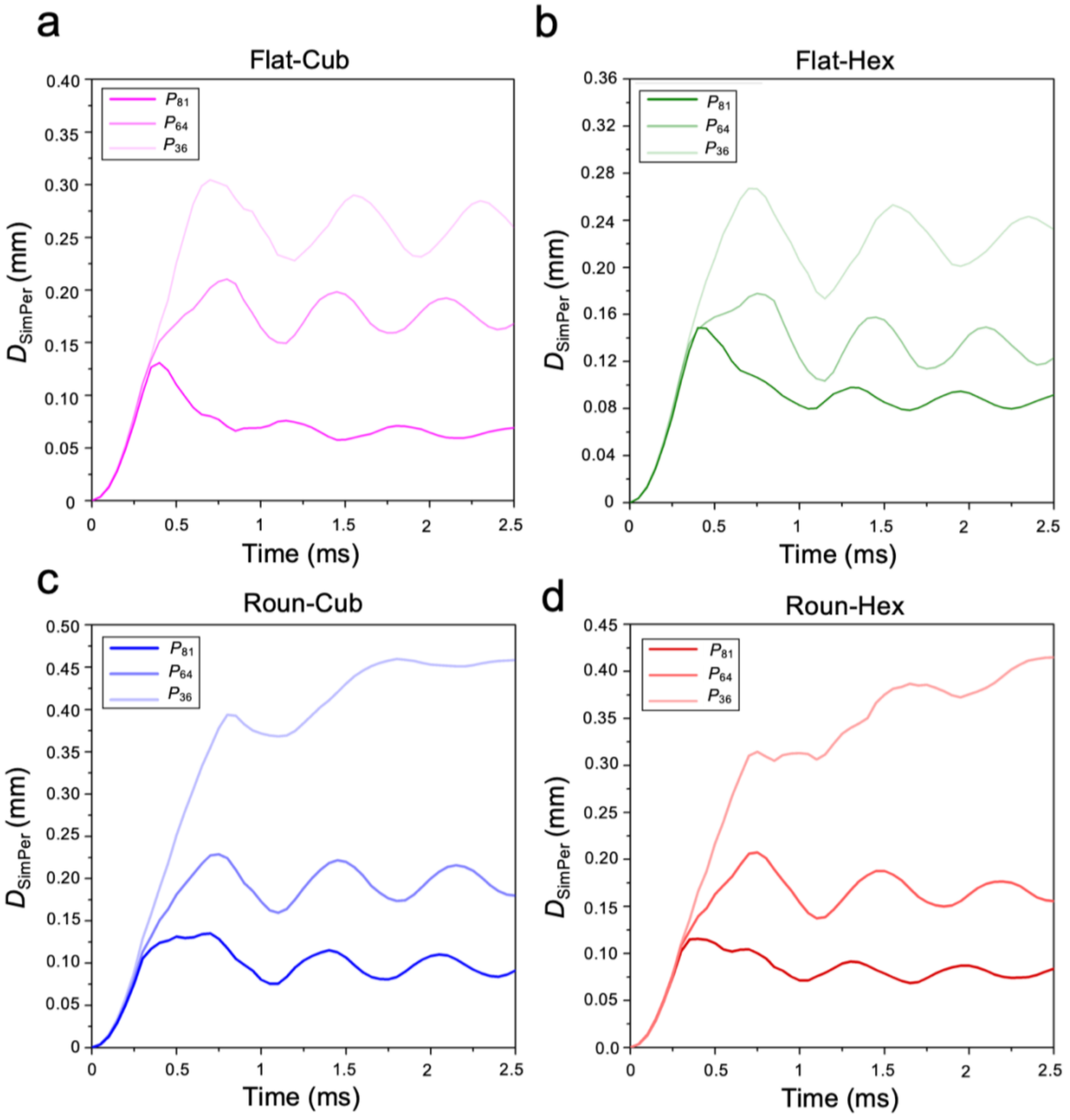

Figure S3: Influence of varying pitch on D_{SimPer} (E_{m}= 2.05MPa) for different MAP designs.

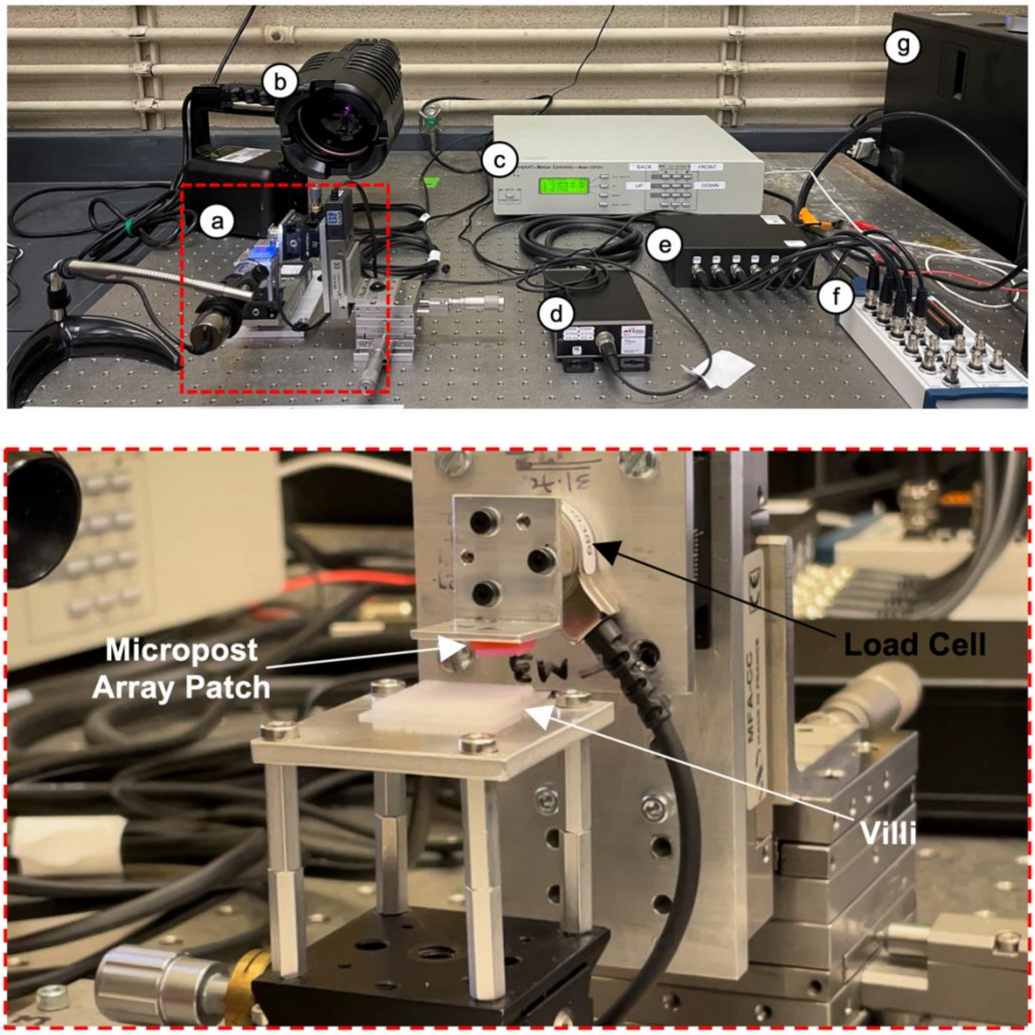

Figure S4: In-vitro lap-shear test bed set-up a) In-vitro test bed. b) UV Lamp. c) ESP301 Motion controller. d) DAQ PS/IFPS Box. e) BNC interface box – 9105-BNC-2. f) Terminal Block with BNC inputs. g) Computer.

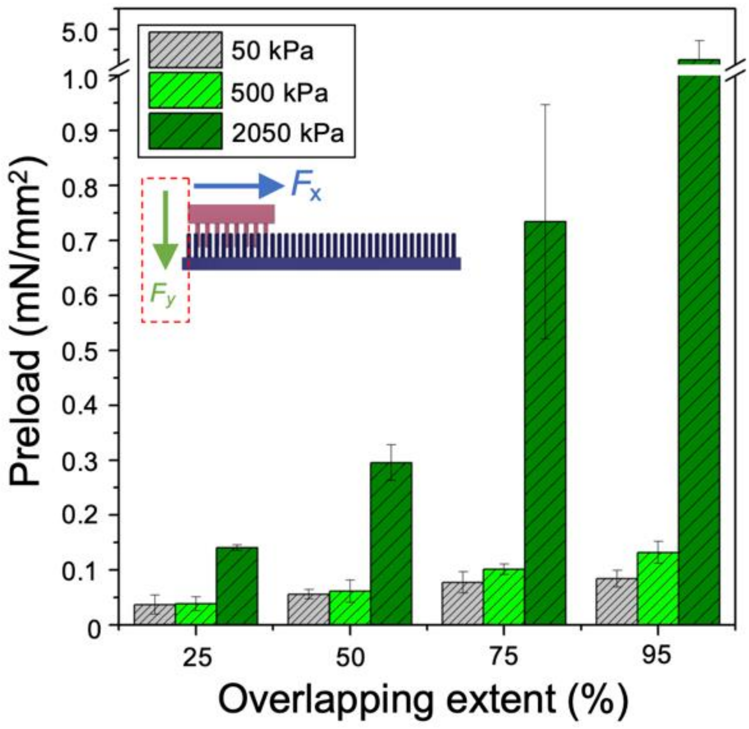

Figure S5: Greater preload on MAPs is required to achieve higher overlap between microposts and villi. Softer MAPs ($E_{\mathrm{m50/500kPa}}$) can achieve 95% overlap with preload under 0.2mN mm^{-2} i.e., ~10 times less than the force exerted by jejunal contractile pressure (2.4 mN mm^{-2}).

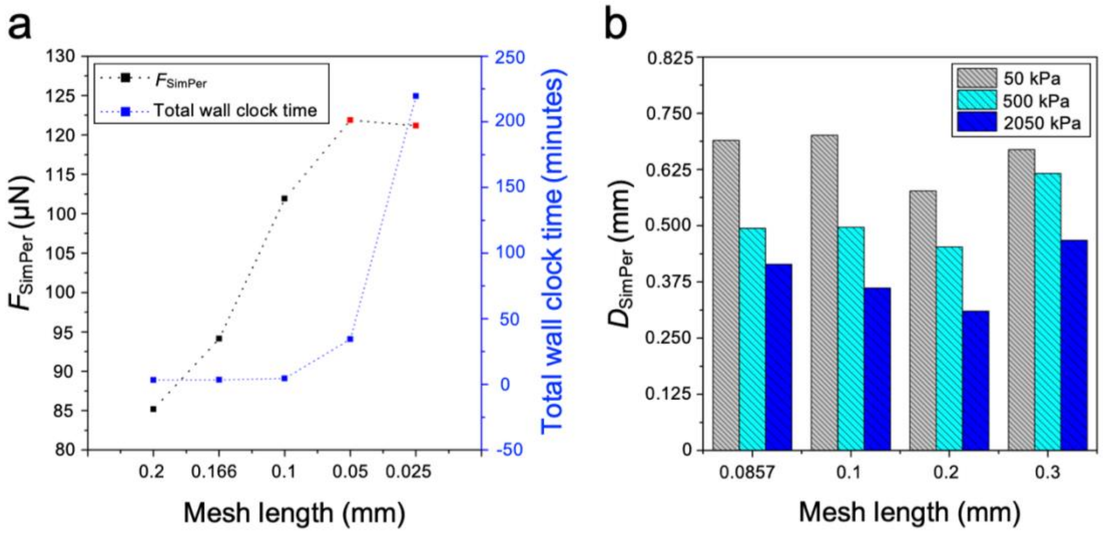

Figure S6: Mesh convergence analyses of MP-V and MAP-VP models a) Parabolic mesh elements (25,552) with a mesh length of 0.05mm were used for calculations. b) Linear mesh elements (263,052) with a mesh length of 0.0857mm were used for calculations to optimize the computation time. Here, change in mesh size did not influence the trends observed.

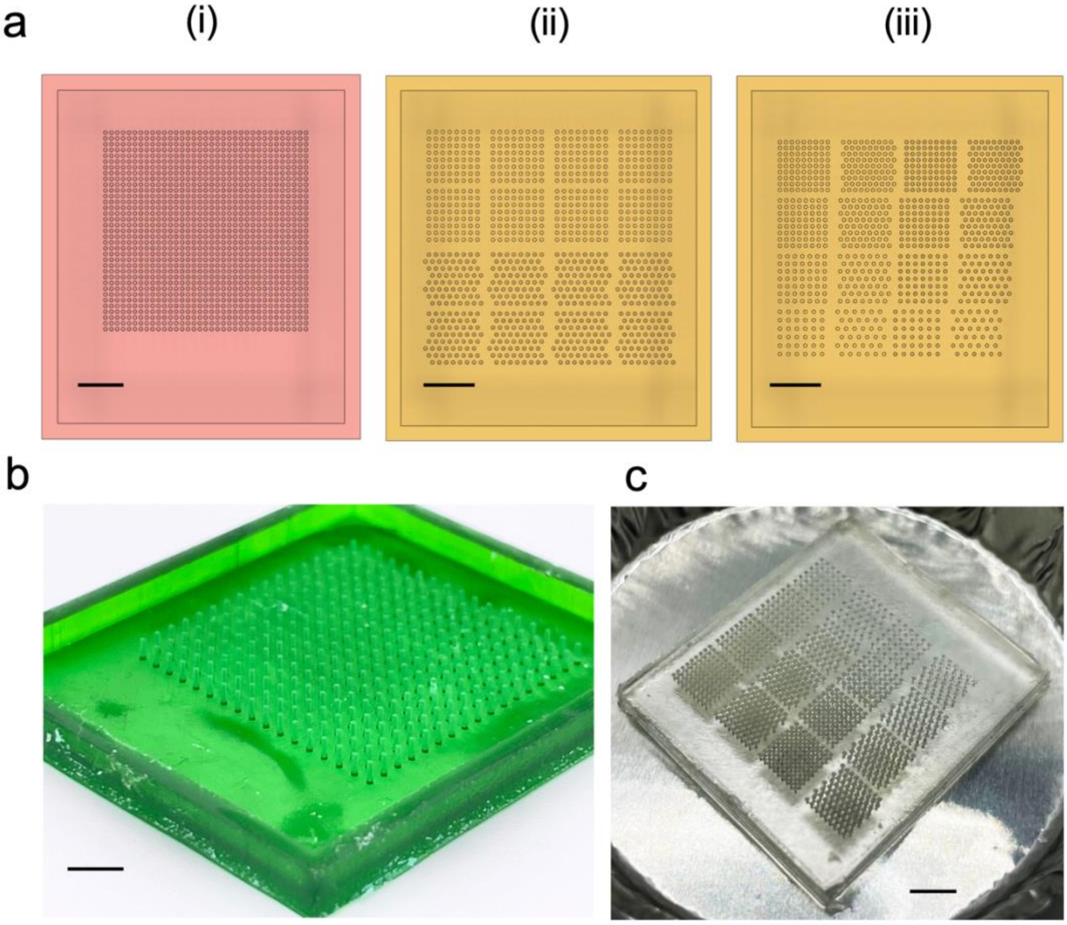

Figure S7: Fabrication of microposts a) STL file prepared for DLP 3D printing the positive molds. (i) Schematic of villi mold (200µm edge-to-edge spacing). (ii) Schematic of MAPs with edge-to-edge spacing of 350µm with flat tipped microposts arranged cubically and round tipped microposts arranged hexagonally. (iii) MAPs with varying spacing (250-500µm), arrangement and tip geometry. (b) 3D printed positive mold, UV cured, washed in IPA, and air-dried. (c) Replica molded negative mold prepared with Sylgard-184 post silanization (Scale bars= 5mm).

Table S1: Summary of Current Technologies for Intestinal Retentive Systems

Type	Examples	Materials/Structure	Intestinal adhesion/retention performance
Chemo-adhesive systems	1. Insulin PPS Patches[1]	Eudragit EPO, Pectin, sodium carboxymethyl cellulose	0.98mNmm^{-2} (shear adhesion)
	2. Self-uncoiling stents[2]	Polyester cylinders covered with mucoadhesive patches	Total retention time ~36 hours
	3. Nanoparticle-assembled bioadhesive coacervate coating[3]	Catechol functionalized end groups with Polyethylene glycol Hydrophilic chains	Work of adhesion 7.07µJmm^{-2}<br><br>Retention time ~2 days
	4. Synthetic epithelium lining[4]	Tissue surface initiated Polydopamine coating	Qualitative analysis of adhesion by scrapping<br><br>Retention time limited to mucus turnover (~12 hours)
Magnetic systems	5. Magnetic living hydrogels[5]	Polyvinyl Alcohol hydrogel matrix doped with NdFeB ferromagnetic microparticles	In-vivo retention 7 days with wearable magnet intact, 6 hours without magnet
	6. Polymeric microparticles[6]	core–shell chitosan–alginate Fe_3O_4 incorporated magnetic beads	Tested for 2 hours
	7. Charge-coupled polymeric microparticles and micromagnets[7]	PLGA based microparticles, Superparamagnetic iron oxide microparticles	Retention time with magnet ~36 hours
Mechano-adhesives	8. Swellable microneedles – biphasic microneedle with swellable tips[8]	Swelling of proboscis of Pomphorhynchus laevis	Mean adhesion strength 4.53Ncm^{-2}
	9. Barbed microneedles – tissue anchoring microneedles[9]	Spikes of proboscis of spiny-head worm Acanthocephala	Max pull-out force 25mN per Microneedle
	10. Tissue attachment mechanism[10]	Sucker teeth of leech and tapeworms	Max adhesion strength 8.09N per device<br><br>Retention time 6 days
	11. Microfibrillar adhesives – Van der Waals forces and capillary action[11]	Microtrichia of beetle wings and oily secretions	Max adhesion 18.5mNmm^{-2} for preload 20mNmm^{-2} diameter 4µm (HAR) (shear)
Bio-inspired mechanical interlocking unexplored for intestinal adhesion	12. Suction cups – Capillary action[12]	Octopus suckers	1.8Ncm^{-2} with skin tissues
	13. Microhooks – abaxial and adaxial hooks[13]	Climbing plant hooks Galium Aparine leaf	Shear anchoring force on Artificial skin at preload 1N – ~7.5N cm^{-2}
	14. Micro/nano hairs – shear mechanical interlocking[14]	Wing-locking mechanism of Beetle wings via microtrichia (Van der Waals interactions)	Shear adhesion (preload 1N) between hair patches AR 10)<br>Nanohairs (d 100um) ~40N cm^{-2}<br>Microhairs (d 6µm) ~10N cm^{-2}
Friction enhancement	15. Micro-patterned wet adhesives (aspect ratio ~1:1) [15]<br><br>Incorporated in tri-legged capsule anchoring system[16] and active capsule endoscopy[17]		0.0828Ncm^{-2}<br>(Preload 0.131N, height 125µm, diameter 140µm, edge-to-edge spacing 105µm, viscosity 10000 cSt)

Table S2: Adjusted length of 3D printed villi after UV curing (*n*=5)

Initial length (mm)	Length after curing (mm)	Percent difference
1.75	1.338 ± 0.01	– 10.8
1.8	1.39 ± 0.005	– 7.34
1.86	1.496 ± 0.015	– 0.245
1.9	1.584 ± 0.027	+ 5.56
2.0	1.584 ± 0.0123	+ 5.56
2.25	1.805 ± 0.0250	+ 20.34
2.5	2.026 ± 0.014	+ 35.08

Table S3: Adjusted length of 3D printed MAP microposts after UV curing (*n*=5)

Initial length (mm)	Length after curing (mm)	Percent difference
1.75	1.307 ± 0.03	– 12.867
1.85	1.300 ± 0.01	– 13.289
1.95	1.484 ± 0.009	– 1.045
2	1.477 ± 0.009	– 1.56
2.1	1.60 ± 0.001	+ 6.689
2.25	1.597 ± 0.006	+ 6.45
2.5	1.847 ± 0.02	+ 23.1

The quality of printed microposts in MAP molds was also influenced by the distance between individual patches. The adjusted gaps between them were 1.565mm (lateral) and 1.065mm (longitudinal). These gaps allowed ease of slicing MAPs into desired squares with cross-sectional area of 25mm^2.

Table S4: PDMS formulations to prepare microposts of required moduli.

Young's moduli (kPa)	PDMS type	Curing temperature (°C)	Curing time (minutes)
50	Ecoflex-0010	80	120
500	Ecoflex-0010: Sylgard-184	80	120
2050	Sylgard-184	100	40

Additional References

[1] A. Banerjee, J. Lee, S. Mitragotri, *Bioengineering & Translational Medicine* **2016**, *1*, 338.
[2] S. Sarker, R. Jones, G. Chow, B. Terry, in *2021 Design of Medical Devices Conference*, American Society Of Mechanical Engineers, Minneapolis, MN, USA, **2021**, p. V001T12A010.
[3] P. Zhao, X. Xia, X. Xu, K. K. C. Leung, A. Rai, Y. Deng, B. Yang, H. Lai, X. Peng, P. Shi, H. Zhang, P. W. Y. Chiu, L. Bian, *Nat Commun* **2021**, *12*, 7162.
[4] J. Li, T. Wang, A. R. Kirtane, Y. Shi, A. Jones, Z. Moussa, A. Lopes, J. Collins, S. M. Tamang, K. Hess, R. Shakur, P. Karandikar, J. S. Lee, H.-W. Huang, A. Hayward, G. Traverso, *Sci. Transl. Med.* **2020**, *12*, eabc0441.
[5] X. Liu, Y. Yang, M. E. Inda, S. Lin, J. Wu, Y. Kim, X. Chen, D. Ma, T. K. Lu, X. Zhao, *Adv. Funct. Mater.* **2021**, *31*, 2010918.
[6] A. Seth, D. Lafargue, C. Poirier, J.-M. Péan, C. Ménager, *European Journal of Pharmaceutics and Biopharmaceutics* **2014**, *88*, 374.
[7] B. A. Teply, R. Tong, S. Y. Jeong, G. Luther, I. Sherifi, C. H. Yim, A. Khademhosseini, O. C. Farokhzad, R. S. Langer, J. Cheng, *Biomaterials* **2008**, *29*, 1216.
[8] S. Y. Yang, E. D. O'Cearbhaill, G. C. Sisk, K. M. Park, W. K. Cho, M. Villiger, B. E. Bouma, B. Pomahac, J. M. Karp, *Nat Commun* **2013**, *4*, 1702.
[9] S. Liu, S. Chu, G. E. Banis, L. A. Beardslee, R. Ghodssi, in *2020 IEEE 33rd International Conference on Micro Electro Mechanical Systems (MEMS)*, IEEE, Vancouver, BC, Canada, **2020**, pp. 885–888.
[10] W. Xie, V. Kothari, B. S. Terry, *Biomed Microdevices* **2015**, *17*, 68.
[11] E. Cheung, M. E. Karagozler, Sukho Park, Byungkyu Kim, M. Sitti, in *Proceedings, 2005 IEEE/ASME International Conference on Advanced Intelligent Mechatronics.*, IEEE, Monterey, CA, **2005**, pp. 551–557.
[12] S. Baik, J. Kim, H. J. Lee, T. H. Lee, C. Pang, *Adv. Sci.* **2018**, *5*, 1800100.
[13] I. Fiorello, O. Tricinci, G. A. Naselli, A. Mondini, C. Filippeschi, F. Tramacere, A. K. Mishra, B. Mazzolai, *Adv. Funct. Mater.* **2020**, *30*, 2003380.
[14] C. Pang, D. Kang, T. Kim, K.-Y. Suh, *Langmuir* **2012**, *28*, 2181.
[15] J. Kwon, E. Cheung, S. Park, M. Sitti, *Biomed. Mater.* **2006**, *1*, 216.
[16] P. Glass, E. Cheung, M. Sitti, *IEEE Trans Biomed Eng* **2008**, *55*, 2759.
[17] E. Buselli, V. Pensabene, P. Castrataro, P. Valdastri, A. Menciassi, P. Dario, *Meas. Sci. Technol.* **2010**, *21*, 105802.
